# Comment on Shaniv et al. Neonatal Drug Formularies—A Global Scope. *Children* 2023, *10*, 848

**DOI:** 10.3390/children10111802

**Published:** 2023-11-13

**Authors:** Elisabeth V. Giger, Romy Tilen

**Affiliations:** SwissPedDose, 8032 Zurich, Switzerland; elisabeth.giger@swisspeddose.ch

We read the article by Shaniv D. et al. entitled “Neonatal Drug Formularies—A Global Scope” [1] with great interest. As mentioned in this study, SwissPedDose, the organization that is harmonizing the Swiss national pediatric drug doses, unfortunately missed the opportunity to participate, which we sincerely regret. We would like to briefly explain our process in providing neonatal dosing recommendations based on the questionnaire sent to the participants of the above-mentioned article (see the Supplementary Materials of the article, Document S1).

The Swiss database for dosing medicinal products in pediatrics is an online database (www.swisspeddose.ch/database (accessed on 26 September 2023)) containing nationally harmonized dosage recommendations for neonates and children. It is intended for health care professionals and is free of charge [2]. Age categories can be chosen to distinguish between children and newborn dosing recommendations that are published in German, French and English. Institutional users can download the complete data set as an XML file and integrate the SwissPedDose dosage recommendations directly into their respective clinical information systems with a free subscription. The development of the formulary is based on a legal basis [3] and is therefore financially supported by the Federal Office of Public Health. Experts from the largest children’s hospitals in Switzerland are actively participating in the development of the formulary.

Drugs are chosen by asking the senior pediatricians involved in the dosage-finding process about the requirements for clinical practice. The cases for the selected substances are prepared using a standardized process and cover the relevant indications and routes of administration. In-house dosage data provided by hospital pharmacists of the participating children’s hospitals, drug labeling in Switzerland and/or from a country with similar standards of drug approval (if available), information found in recognized international and national referenced handbooks and online databases such as the British National Formulary for Children (BNFc), uptodate, kinderformulariumn.nl and NNF8, together with results from a literature search, are all considered for this process. This PubMed search consists of keywords relating to aspects such as substance, indication, route of administration and age filter (“newborn: birth-1 month”). All the literature consulted in the process is listed for each case. Age and weight are specified where needed: current weight, birth weight, gestational age (GA), postmenstrual age (PMA) and postnatal age (PNA).

The neonatal formulary cases are revised and discussed with senior neonatologists from nine Swiss children’s hospitals. Only if a consensus among the experts can be reached is the case published as the national neonatal drug dose. If there are major concerns among the experts about a dosage recommendation, a research question for SwissPedNet, the Swiss Research Network of Clinical Pediatric Hubs, is formulated.

At least every four years, dosage recommendations are revised in a defined re-evaluation process taking the most recent literature into consideration. Feedback from users provided directly via the feedback button or new evidence found by anyone involved in the process can lead to immediate changes. Subscribers are informed via an automatic email about updates, while all users can find updates on the changelog page. At the time of writing, 157 dosage recommendations for 88 substances are available in the Swiss neonatal formulary. More information about the formulary is available upon request from the authors.

We would like to thank the authors of “Neonatal Drug Formularies—A Global Scope” for their important work and for their invitation to describe our process in this comment.
